# Diacylglycerol kinase alpha is a proliferation marker of intrahepatic cholangiocarcinoma associated with the prognosis

**DOI:** 10.1002/cam4.7238

**Published:** 2024-05-08

**Authors:** Shunsuke Shichi, Ko Sugiyama, Yoh Asahi, Chisato Shirakawa, Hiroki Nakamoto, Saori Kimura, Kazuki Wakizaka, Takeshi Aiyama, Akihisa Nagatsu, Tatsuya Orimo, Tatsuhiko Kakisaka, Akinobu Taketomi

**Affiliations:** ^1^ Department of Gastroenterological Surgery I Hokkaido University Graduate School of Medicine Sapporo Japan

**Keywords:** biomarker, diacylglycerol kinase, intrahepatic cholangiocarcinoma, primary liver cancer, prognostic factor

## Abstract

**Background:**

Intrahepatic cholangiocarcinoma (ICC) has a high recurrence rate and a poor prognosis. Thus, the development of effective treatment and prognostic biomarkers is required. High expression of diacylglycerol kinase alpha (DGKα) is a prognostic factor for the recurrence of hepatocellular carcinoma. However, the relationship between DGKα expression and prognosis in ICC has not been reported.

**Methods:**

Immunohistochemistry (IHC) with anti‐DGKα antibody was performed on surgical specimens of ICC (*n* = 69). First, DGKα expression in cancer cells was qualitatively classified into four groups (−, 1+, 2+, 3+) and divided into two groups (DGKα− and DGKα+1 + to 3+). The relationship between clinical features and DGKα expression was analyzed. Second, Ki‐67 expression was evaluated as a cell proliferation marker. The number of Ki‐67‐positive cells was counted, and the relationship with DGKα expression was examined.

**Results:**

DGKα IHC divided the patients into a DGKα+ group (1+: *n* = 15; 2+: *n* = 5; 3+: *n* = 5) and a DGKα− group (−: *n* = 44). In the DGKα+ group, patients were older and had advanced disease. Both overall survival and recurrence‐free survival (RFS) were significantly worse in the DGKα+ patients. DGKα+ was identified as an independent prognostic factor for RFS by multivariate analysis. Furthermore, the number of Ki‐67‐positive cells increased in association with the staining levels of DGKα.

**Conclusion:**

Pathological DGKα expression in ICC was a cancer proliferation marker associated with recurrence. This suggests that DGKα may be a potential therapeutic target for ICC.

## 1 INTRODUCTION

Primary liver cancer is one of the most intractable gastrointestinal tumors and most cases are hepatocellular carcinoma (HCC), but the next most common histological type is intrahepatic cholangiocarcinoma (ICC). ICC is a type of cholangiocarcinoma that arises peripherally from the secondary branches. In Japan, it is present in approximately 6% of primary liver cancers,^1^ and the 5‐year overall survival (OS) rate after surgery is reported to be 31%–42.2%.^2^, ^3^, ^4^ The incidence is increasing worldwide, including in Japan,^1^ but the cause has not been identified. Although surgery is the first‐line curative treatment, the resection rate at diagnosis is less than 60%^5^, ^6^ and the postoperative recurrence rate is more than 70%,^7^, ^8^, ^9^ and no effective nonsurgical treatment such as drug therapy has yet been established. Against this background, there is an urgent need to identify effective treatments and biomarkers to improve the poor prognosis of this disease.

DGKα has been reported as a prognostic factor involved in the growth of HCC and is considered to be an upstream factor of Raf–MEK–ERK in HCC and amplifies targets downstream of MET.^10^ Indeed, DGKα inhibitors in vitro and in vivo could suppress cancer cell proliferation.^11^, ^12^, ^13^ Inhibition of DGKα induces apoptosis in DGKα‐expressed cancer cell lines regardless of their origins.^12^ So, DGKα has been thought of as an important cell growth factor in cancers.

On the other hand, DGKα has been reported as a key molecule inducing the anergy of T cells.^14^ Using a liver tumor model, it has been shown that DGKα inhibitors exert their antitumor effects via T cell immunostimulant,^11^ and basic studies have shown that inhibitors better inhibit cell proliferation in multiple cancer cell lines with DGKα expression.^12^ DGKα inhibition is considered a novel cancer therapeutic target.^15^

Regarding the evaluation of immunostaining in human cancer tissues, there is a report related to the prognosis of colorectal cancer (CRC) patients. CRC stage II (pT3N0M0) patients who highly expressed DGKα in cancer cells and stromal cells that were considered equal to tumor‐infiltrating lymphocytes had a worse prognosis.^13^ In this study, we investigated the relationship between DGKα expression and the clinicopathological features and prognosis of ICC.

## 2 METHODS

### 2.1 Patient selection and clinical treatment

Seventy ICC patients underwent hepatectomy at Hokkaido University Hospital from 1997 to 2013. Among these, the histopathology and clinical outcomes of 69 patients were analyzed; 1 case was excluded due to a lack of appropriate pathological specimens. Patients who were diagnosed with ICC preoperatively underwent hepatectomy and lymph node dissection of the hepatoduodenal ligament and the area around the common hepatic artery and retro pancreas head. When the tumor was located in the left lobe, the lymph nodes around the lesser curvature of the stomach were also dissected. When the tumor had bile duct invasion, the extrahepatic bile duct was resected and biliary reconstruction was performed. Patients from 2000 to 2007 were treated with 5‐fluorouracil‐based adjuvant chemotherapy, and patients after 2007 were treated gemcitabine‐based adjuvant chemotherapy. Patients were followed up every 3 months until 5 years after surgery. The maximum follow‐up was 13 years, and there were 5 patients whose hospital visits were discontinued within 5 years for reasons other than death.

### 2.2 Immunohistochemical staining

An original anti‐DGKα monoclonal antibody named DaMab‐8 was produced by Sano et al.^16^ and donated from our collaborator, Ono Pharmaceutical Co., Ltd. Four‐micrometer‐thick sections of formalin‐fixed, paraffin‐embedded specimens were used for immunohistochemical staining. After deparaffinization, antigen retrieval was performed using a citric acid buffer and heated for 20 min at 95°C, and endogenous peroxidase activity was blocked with 0.3% hydrogen peroxide at room temperature for 10 min. Sections were washed with Tris‐buffered saline and then incubated with antihuman DGKα monoclonal antibodies (DaMab‐8, 1:500) overnight at 4°C, or with anti‐Ki‐67 monoclonal antibodies (ab16667, Abcam), followed by incubation at room temperature for 30 min with Histofine Simple Stain, MAX PO (MULTI). Proteins were visualized using 3–3′‐diaminobenzidine‐4HCL at room temperature for 5 min, followed by counterstaining with Mayer's hematoxylin.

### 2.3 Evaluation

DGKα staining in cancer cells was evaluated at four levels according to the intensity of staining. Patients were classified as stain positive when the cytoplasm of cancer cells was recognized as positively stained in the high‐power field of view and were classified into four levels according to their staining intensity. In all samples, there were peritumoral inflammatory cells like lymphocytes that were positive for staining, and the staining level was confirmed to be the same. Ki‐67 staining was calculated as the percentage of positively stained cells out of the total number of cancer cells in each of the four intensely magnified fields. Cell counts were performed using ImageJ version: 2.1.0/1.53c. At least 500 cancer cells per patient were included in the field of view. The scores were evaluated by two different medical doctors in a double‐blind manner, and the lower scores were used if the score differed depending on the evaluator.

### 2.4 Statistical analysis

Comparisons of the relationship between background factors and DGKα were made using Fisher's exact test for categorical variables and the Mann–Whitney.

*U* test for continuous variables. Survival curves were estimated using the Kaplan–Meier method, and the differences in survival rates between groups were compared by the log‐rank test. Univariate and multivariate analyses were performed using Cox's proportional hazards regression model to evaluate independent factors predictive of patient survival. Multivariate analysis was performed using the factors extracted in the univariate analysis. In analyses related to recurrence‐free survival (RFS), cases with curability C were excluded.

When DGKα was classified into positive and negative groups, the Mann–Whitney *U* test was used for comparison with the Ki‐67‐positive cells. However, when DGKα was classified according to staining intensity, the Kruskal–Wallis test was used followed by multiple comparisons using the Steel–Dwass test. *p*‐Values of <0.05 were considered to be significant.

All statistical analyses were performed using EZR (Saitama Medical Center, Jichi Medical University, Saitama, Japan) as a graphical user interface for R (The R Foundation for Statistical Computing, Vienna, Austria).

## 3 RESULTS

### 3.1 Evaluation method of DGKα immunohistochemistry in cases of ICC

**FIGURE 1:**
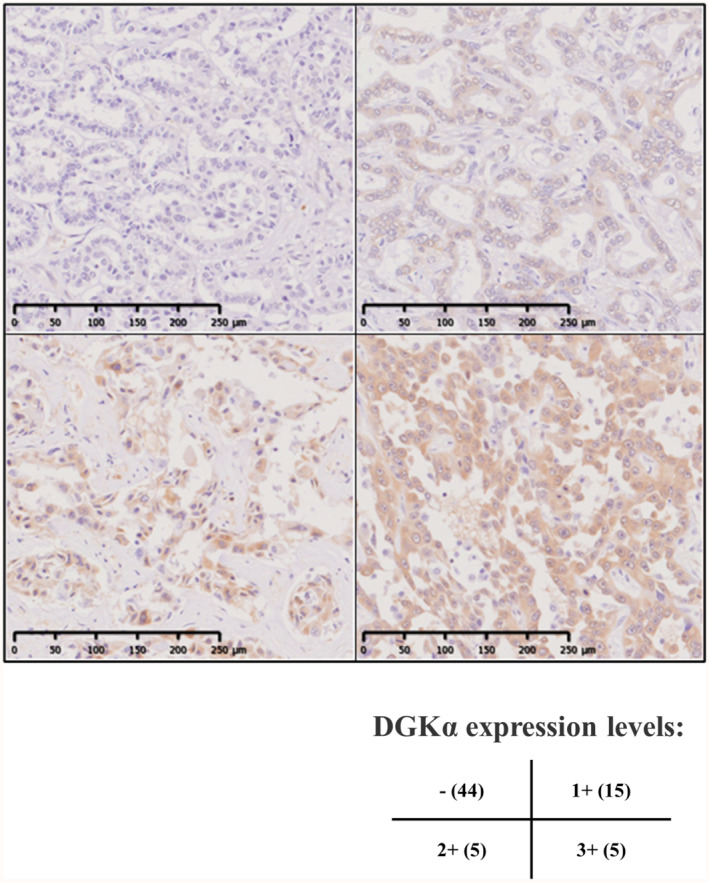
Representative images of immunohistochemical staining of diacylglycerol kinase alpha (DGKα). DGKα expression levels were divided into four groups (−, 1+, 2+, and 3+).

Patients' backgrounds are shown in Table 1. DGKα staining of cancer tissues from the patients was classified into three levels according to the staining intensity in the cancer cell cytoplasm, and cases in which cancer cells in the sections could not be judged to be specifically stained were defined as negative for staining. Representative images of immunohistochemical staining for DGKα are shown in Figure 1. Forty‐four cases were negative (−), 15 cases were weakly positive (1+), 5 cases were moderately positive (2+), and 5 cases were strongly positive (3+).

**TABLE 1:** Clinicopathological characteristics of 69 ICC patients who underwent surgery at Hokkaido University Hospital from 1997 to 2013.

	*n* = 69
Age	
<65	40
65≤	29
Sex	
Male	42
Female	27
Adjacent liver	
Normal	51
Others	18
HBV and/or HCV infection	
−	51
+	18
CA19‐9 (U/mL)	
≤37	23
37<	41
Unknown	5
Tumor size (cm)	
<5	29
5≤	40
Tumor number	
Single	51
Multiple	18
Pathological differentiation	
Well or moderately	40
Poorly	29
Lymph node metastasis	
−	40
+	29
Surgical procedure (resected segments)	
<2	11
2≤	58
Vascular invasion	
−	54
+	15
Pathological stage	
2 or 3	30
4	39
Curability	
A or B	48
C	21

Abbreviation: ICC, Intrahepatic cholangiocarcinoma.

### 3.2 Clinicopathological features of DGKα‐positive cases

In the DGKα‐positive group (1+ to 3+), patients were older and had higher CA19‐9 levels and more advanced disease. There were no differences in tumor size, number of tumors, tumor differentiation, lymph node metastasis, degree of vascular invasion, and surgical factors (resection volume and curability) between the DGKα‐positive and ‐negative groups (Table 2).

**TABLE 2:** Clinicopathological characteristics according to DGKα expression.

	DGKα		*p*‐Value
	Negative	Positive	
Age			
<65	32	10	0.0413
65≤	14	15	
Sex			
Male	28	16	0.799
Female	18	9	
Adjacent liver			
Normal	36	16	0.169
Others	10	8	
HBV and/or HCV infection			
−	35	17	0.409
+	11	8	
CA19‐9 (U/mL)^†^	44.1 [0.0, 3611.7]	110.7 [0.0, 97749.7]	0.044
Tumor size (cm)^†^	5.2 [1.4, 17.0]	6.4 [2.4, 18.0]	0.126
Tumor number			
Single	33	18	0.783
Multiple	11	7	
Pathological differentiation			
Well or moderately	29	11	0.127
Poorly	17	14	
Lymph node metastasis			
−	29	11	0.127
+	17	14	
Surgical procedure (resected segments)			
<2	8	3	0.734
2≤	36	22	
Vascular invasion			
−	12	4	0.546
+	34	21	
Pathological stage			
2 or 3	24	6	0.0223
4	22	19	
Curability			
A or B	35	14	0.101
C	11	11	

Abbreviation: DGKα, diacylglycerol kinase alpha.

^†^ Median [minimum, maximum].

### 3.3 DGKα‐positive patients have poorer OS and RFS

**FIGURE 2:**
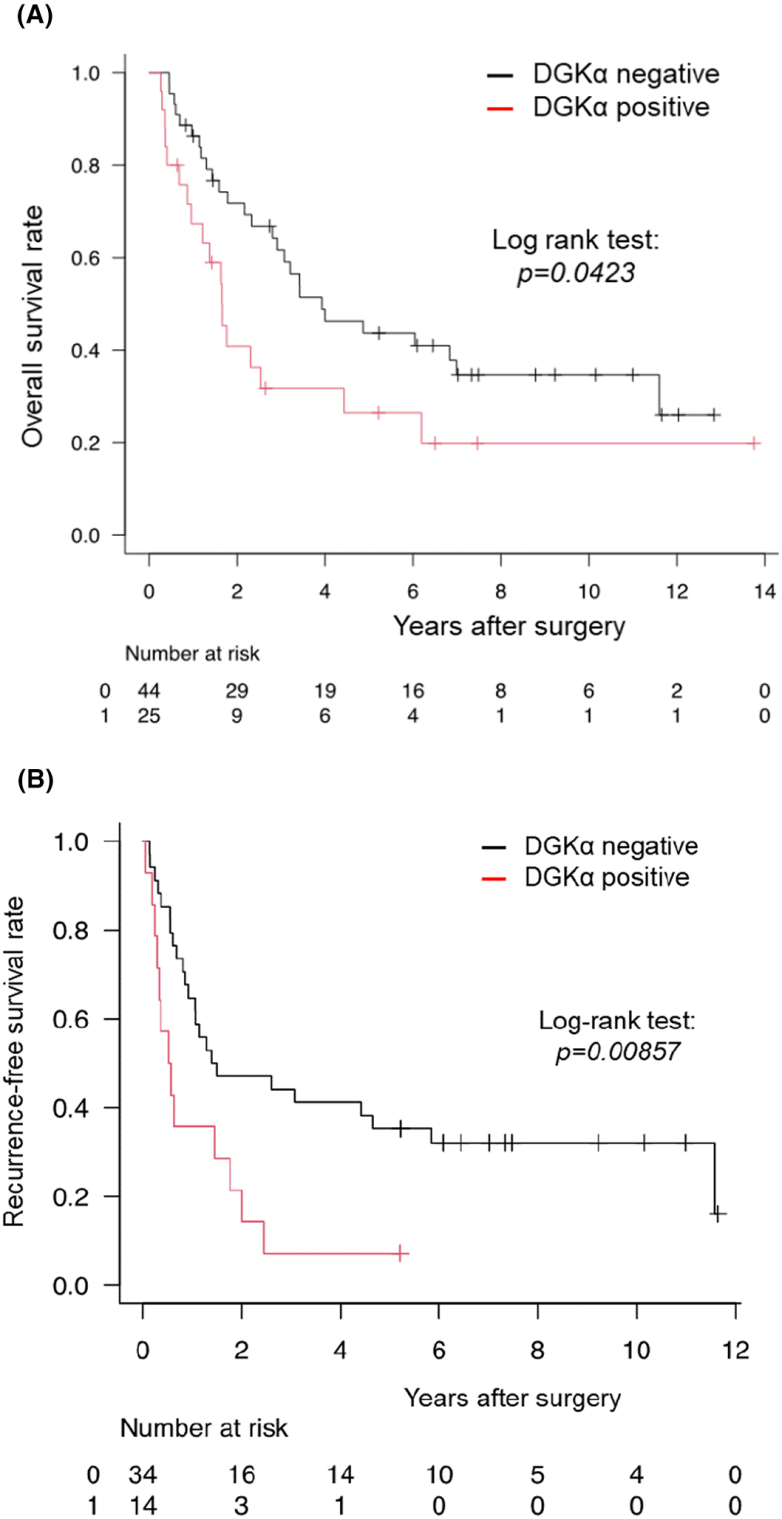
Kaplan–Meier curve of diacylglycerol kinase alpha (DGKα) expression for overall survival (A) and recurrence‐free survival (B), divided into two groups: DGKα‐negative (−) and DGKα‐positive (1 +–3+).

Survival time analysis was performed and Kaplan–Meier curves are shown in Figure 2. In the log‐rank test, OS at 5 years after surgery was *p* = 0.0423 (Figure 2A) and RFS was *p* = 0.00857 (Figure 2B). In both cases, the DGKα‐positive group had a significantly poor prognosis.

### 3.4 DGKα positivity is an independent poor prognostic factor for RFS in multivariate analysis

Regression analysis of each clinicopathological factor showed that, for OS (Table 3), the prognostic factors identified in the univariate analysis were sex, number of tumors, lymph node metastasis, vascular invasion, pathological stage, and surgical curability. In the multivariate analysis, sex, number of tumors, lymph node metastasis, vascular invasion, and surgical radiosurgery were independent prognostic factors. For RFS (Table 4) analyzed excluding curability C, hepatitis virus infection, tumor number, pathological stage, and DGKα positivity were prognostic factors in the univariate analysis. In the multivariate analysis, tumor number and DGKα positivity were independent prognostic factors.

**TABLE 3:** Univariate and multivariate analyses of prognostic factors related to overall survival.

OS	Univariate			Multivariate		
Variable	HR	95% CI	*p*‐Value	HR	95% CI	*p*‐Value
Age (65≤/<65)	1.274	0.706–2.301	0.4217			
Sex (male/female)	1.903	1.006–3.599	0.0480	3.628	1.794–7.336	0.0003
Adjacent liver (normal/others)	1.562	0.828–2.946	0.1683			
HBV or HCV (positive/negative)	1.309	0.702–2.444	0.397			
CA19‐9 (37</≤37) (U/mL)	1.494	0.784–2.855	0.2249			
Tumor size (5≤/<5) (cm)	1.783	0.947–3.356	0.0732			
Tumor number (multiple/single)	2.207	1.164–4.182	0.0152	3.078	1.512–6.265	0.0019
Pathological differentiation (well or moderately/poorly)	0.751	0.418–1.348	0.3374			
Lymph node metastasis (positive/negative)	2.069	1.139–3.756	0.0169	2.540	1.344–4.798	0.0041
Surgical procedure (resected areas) (2≤/<2)	1.384	0.639–2.999	0.4104			
Vascular invasion (positive/negative)	2.190	1.003–4.780	0.0490	2.747	1.194–6.321	0.0175
pStage (4/3 or 2)	3.010	1.581–5.731	0.0008			
Curability (C/A or B)	2.125	1.148–3.936	0.0165	2.777	1.363–5.659	0.0049
DGKα (positive/negative)	1.850	1.013–3.381	0.0454	1.656	0.864–3.177	0.1289

Abbreviations: CI, confidence interval; DGKα, diacylglycerol kinase alpha; HR, hazard ratio; OS, overall survival.

**TABLE 4:** Univariate and multivariate analyses of prognostic factors related to recurrence‐free survival.

RFS	Univariate			Multivariate		
Variable	HR	95% CI	*p*‐Value	HR	95% CI	*p*‐Value
Age (65≤/<65)	0.8925	0.462–1.723	0.7346			
Sex (male/female)	1.528	0.749–3.120	0.2441			
Adjacent liver (normal/others)	1.873	0.929–3.773	0.07924			
HBV or HCV (positive/negative)	2.114	1.65–4.200	0.03247	1.428	0.682–2.991	0.3450
CA19‐9 (37</≤37) (U/mL)	1.463	0.729–2.936	0.2846			
Tumor size (5≤/<5) (cm)	1.668	0.866–3.213	0.1265			
Tumor number (multiple/single)	2.654	1.331–5.291	0.005559	3.173	1.472–6.838	0.003208
Pathological differentiation (well or moderately/poorly)	0.6722	0.350–1.291	0.2331			
Lymph node metastasis (positive/negative)	1.327	0.670–2.629	0.4166			
Surgical procedure (resected areas) (2≤/<2)	1.354	0.592–3.098	0.4732			
Vascular invasion (positive/negative)	1.952	0.887–4.294	0.09629			
pStage (4/3 or 2)	2.521	1.274–4.989	0.007909			
DGKα (positive/negative)	2.483	1.232–5.003	0.01098	3.112	1.450–6.680	0.003579

Abbreviation: CI, confidence interval; DGKα, diacylglycerol kinase alpha; HR, hazard ratio.

### 3.5 DGKα expression intensity correlates with the number of Ki‐67‐positive cells

**FIGURE 3:**
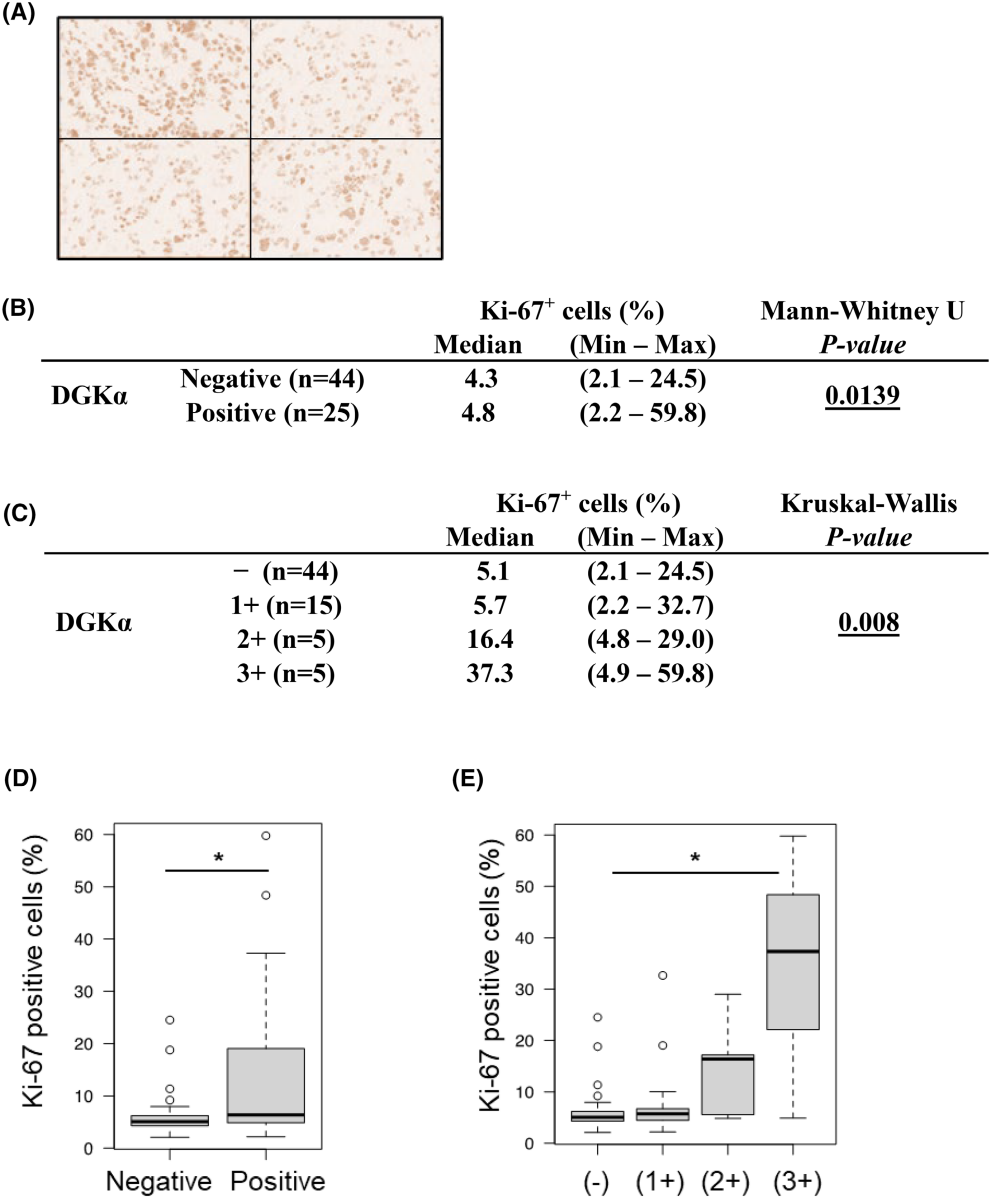
Correlation between diacylglycerol kinase alpha (DGKα) intensity and percentage of Ki‐67‐positive cells. Representative images of one case: four photos in high power field used for counting Ki‐67‐positive cells (A). Ki‐67 positivity was significantly higher in the DGKα‐positive group by Mann–Whitney *U* test (B, D). As DGKα expression levels got higher, Ki‐67 positivity became significantly higher (Kruskal–Wallis test and Steel–Dwass test) (C, E). *p*‐Values are shown as*p < 0.05 in the figures.

Ki‐67 staining was performed using sections of tissue taken near the tissue used for DGKα staining. A representative image is shown in Figure 3A. The number of positively stained cells was evaluated, and the number of Ki‐67‐positive cells was predominantly increased in the DGKα‐positive group (Figure 3B,D), while the number of Ki‐67‐positive cells was higher in the group with higher DGKα intensity (Figure 3C,E). Because Ki‐67 staining positivity in cancer cells is known to be a proliferation marker, increased DGKα expression in ICC may contribute to the proliferation of ICC.

## 4 DISCUSSION

In patients with ICC in this study, those with positive DGKα expression were older and at an advanced pathological stage, and had higher CA19‐9 levels. They had a significantly poorer prognosis regarding both OS and RFS. In the multivariate analysis of prognostic factors in RFS, high DGKα expression was an independent poor prognostic factor. Furthermore, DGKα expression intensity was correlated with the positive rate of Ki‐67 cells and was found to be a factor associated with cancer cell proliferation.

It has been reported that DGKα inhibition induces cancer‐cell apoptosis.^11^, ^12^, ^17^ A DGKα‐specific inhibitor was more effective in inhibiting proliferation in the cell line, which had higher DGKα expression.^12^ Increased DGKα expression may increase the dependence of cancer growth on DGKα function, and pathological evaluation of tumor tissue can indicate the targets of DGKα inhibitory therapy.

It is expected to become a therapeutic target across various cancers from the previous reports. Nevertheless, it has never been investigated DGKα in ICC. This is the first report to investigate the relationship between the pathological DGKα expression level and the clinicopathological features in cholangiocarcinoma.

In addition, in the present study, Ki‐67 staining was evaluated by the previously used evaluation system in HCC cases.^10^ DGKα expression can evaluate cell proliferation regardless of the histological type of primary liver cancer. We have confirmed the antitumor effects of DGKα inhibitors using a mouse model of liver cancer.^11^ These results from human clinical specimens, which revealed similar features in HCC and ICC, provide evidence to optimize the therapeutic indications for DGKα inhibitor therapy in almost all primary liver cancers.

The chemotherapy of biliary tract cancers has advanced rapidly in the past decade. Immune checkpoint inhibitors have also become part of standard therapy,^18^ such as durvalumab and pembrolizumab which inhibit the PD‐1/PD‐L1 pathway. DGKα was reported as a regulator of T cells and DGKα inhibitor is expected as a target of cancer immunotherapy. Our previous research revealed that the antitumor effect of DGKα inhibitor may be synergistically enhanced when used in combination with a PD‐L1 antibody.^11^

In this study, DGKα expression was not an independent factor of OS in the multivariate analysis. This may be because some patients who got noncurative resection received advanced treatments such as chemotherapy or local therapy. Therefore, we consider that the difference was extracted as an independent prognostic factor only concerning RFS.

A limitation of this study is that the data are from a limited number of patients at a single institution. We have not yet found any evidence of DGK expression or activity by other means than tissue staining biomarkers.

We believe that DGKα is a promising biomarker and therapeutic target molecule for primary liver cancer, including not only HCC but also ICC. We hope that the DGK‐related molecular mechanisms will be elucidated and that along with the development of DGKα inhibitory therapy.

## AUTHOR CONTRIBUTIONS

**Shunsuke Shichi:** Conceptualization (lead); data curation (lead); formal analysis (equal); funding acquisition (supporting); investigation (equal); methodology (equal); project administration (equal); resources (equal); software (equal); supervision (supporting); validation (supporting); visualization (supporting); writing – original draft (lead); writing – review and editing (equal). **Ko Sugiyama:** Conceptualization (lead); data curation (lead); formal analysis (equal); funding acquisition (supporting); investigation (equal); methodology (equal); project administration (equal); resources (supporting); software (equal); supervision (equal); validation (equal); visualization (equal); writing – original draft (lead); writing – review and editing (lead). **Yoh Asahi:** Conceptualization (equal); data curation (equal); formal analysis (equal); funding acquisition (equal); investigation (equal); methodology (equal); project administration (equal); resources (equal); software (equal); supervision (equal); validation (equal); visualization (equal); writing – original draft (equal); writing – review and editing (equal). **Chisato Shirakawa:** Conceptualization (equal); data curation (equal); formal analysis (equal); funding acquisition (equal); investigation (equal); methodology (equal); project administration (equal); resources (equal); software (equal); supervision (equal); validation (equal); visualization (equal); writing – original draft (equal); writing – review and editing (equal). **Hiroki Nakamoto:** Conceptualization (equal); data curation (equal); formal analysis (equal); funding acquisition (equal); investigation (equal); methodology (equal); project administration (equal); resources (equal); software (equal); supervision (equal); validation (equal); visualization (equal); writing – original draft (equal); writing – review and editing (equal). **Saori Kimura:** Conceptualization (equal); data curation (equal); formal analysis (equal); funding acquisition (equal); investigation (equal); methodology (equal); project administration (equal); resources (equal); software (equal); supervision (equal); validation (equal); visualization (equal); writing – original draft (equal); writing – review and editing (equal). **Kazuki Wakizaka:** Conceptualization (equal); data curation (equal); formal analysis (equal); funding acquisition (equal); investigation (equal); methodology (equal); project administration (equal); resources (equal); software (equal); supervision (equal); validation (equal); visualization (equal); writing – original draft (equal); writing – review and editing (equal). **Takeshi Aiyama:** Conceptualization (equal); data curation (equal); formal analysis (equal); funding acquisition (equal); investigation (equal); methodology (equal); project administration (equal); resources (equal); software (equal); supervision (equal); validation (equal); visualization (equal); writing – original draft (equal); writing – review and editing (equal). **Akihisa Nagatsu:** Conceptualization (equal); data curation (equal); formal analysis (equal); funding acquisition (equal); investigation (equal); methodology (equal); project administration (equal); resources (equal); software (equal); supervision (equal); validation (equal); visualization (equal); writing – original draft (equal); writing – review and editing (equal). **Tatsuya Orimo:** Conceptualization (equal); data curation (equal); formal analysis (equal); funding acquisition (equal); investigation (equal); methodology (equal); project administration (equal); resources (equal); software (equal); supervision (equal); validation (equal); visualization (equal); writing – original draft (equal); writing – review and editing (equal). **Tatsuhiko Kakisaka:** Conceptualization (equal); data curation (equal); formal analysis (equal); funding acquisition (equal); investigation (equal); methodology (equal); project administration (equal); resources (equal); software (equal); supervision (equal); validation (equal); visualization (equal); writing – original draft (equal); writing – review and editing (equal). **Akinobu Taketomi:** Conceptualization (lead); data curation (equal); formal analysis (equal); funding acquisition (lead); investigation (equal); methodology (lead); project administration (equal); resources (lead); software (equal); supervision (lead); validation (equal); visualization (equal); writing – original draft (equal); writing – review and editing (lead).

## FUNDING INFORMATION

This study was partially supported by Ono Pharmaceutical Co., Ltd. through the donation of research grants and antibody material. This study was also supported by a Grant‐in‐Aid for Scientific Research B (19H03724, 22H03142) and for Challenging Research (Exploratory) (18 K19571, 21 K19516) from the Ministry of Education, Culture, Sports, Science, and Technology, Japan (MEXT), and a grant (22fk0210091h0002) from the Japan Agency for Medical Research and Development (AMED). All funding sources had no involvement in the study design and in the decision to submit the article for publication.

## CONFLICT OF INTEREST STATEMENT

Authors declare no conflict of interest for this article.

## ETHICS STATEMENT

This research was approved by the Institutional Review Board of Hokkaido University Hospital (015‐0379) and performed in compliance with the Declaration of Helsinki. Informed consent from patients was obtained in the form of opt‐out on the website of Hokkaido University Hospital, or written informed consent was obtained from patients. This study did not contain the animal study and research involving recombinant DNA.

## Data Availability

The data that support the findings of this study are available from the corresponding author upon reasonable request.
